# Peroneus brevis split rupture is underreported on magnetic resonance imaging of the ankle in patients with chronic lateral ankle pain

**DOI:** 10.1016/j.ejro.2024.100591

**Published:** 2024-07-18

**Authors:** Katarzyna Bokwa-Dąbrowska, Dan Mocanu, Alex Alexiev, Katarina Nilsson Helander, Pawel Szaro

**Affiliations:** a Department of Radiology, Institute of Clinical Sciences, Sahlgrenska Academy, University of Gothenburg, Gothenburg, Sweden; b Department of Musculoskeletal Radiology, Sahlgrenska University Hospital, Gothenburg, Sweden; c Department of Orthopedics, Institute of Clinical Sciences, Sahlgrenska Academy, University of Gothenburg, Gothenburg, Sweden; d Department of Orthopedics, Sahlgrenska University Hospital, Gothenburg, Sweden

**Keywords:** Ankle, Foot, Tendon, Magnetic resonance imaging, Injury

## Abstract

**Introduction:**

Peroneus brevis split rupture poses a diagnostic challenge, often requiring magnetic resonance imaging (MRI), yet splits are missed in initial radiological reports. However, the frequency of reported peroneus brevis split rupture in clinical MRI examinations is unknown.

**Aim:**

This study aimed to investigate underreporting frequency of peroneus brevis split rupture in patients with lateral ankle pain.

**Methods:**

We re-evaluated 143 consecutive MRI examinations of the ankle joint, conducted in 2021 in our region, for patients experiencing ankle pain persisting for more than 8 months. Two musculoskeletal radiologists, with 12 and 8 years of experience respectively, assessed the presence of peroneus brevis split rupture. Patients with recent ankle trauma, fractures, postoperative changes, or MRI artifacts were excluded. The radiologists evaluated each MRI for incomplete or complete peroneus brevis split rupture. The consensus between the raters was used as the reference standard. Additionally, raters reviewed the original clinical radiological reports to determine if the presence of peroneus brevis split rupture was noted. Agreement between raters' assessments, consensus, and initial reports was evaluated using Gwet’s AC1 coefficients.

**Results:**

Initial radiological reports indicated 23 cases (52.3 %) of peroneus brevis split rupture, meaning 21 cases (47.7 %) were underreported. The Gwet’s AC1 coefficients showed that the agreement between raters and initial reports was 0.401 (standard error 0.070), 95 % CI (0.261, 0.541), p<.001, while the agreement between raters in the study was 0.716 (standard error 0.082), 95 % CI (0.551, 0.881), p<.001.

**Conclusion:**

Peroneus brevis split rupture is underreported on MRI scans of patients with lateral ankle pain.

## Graphical Abstract

**Figure:**
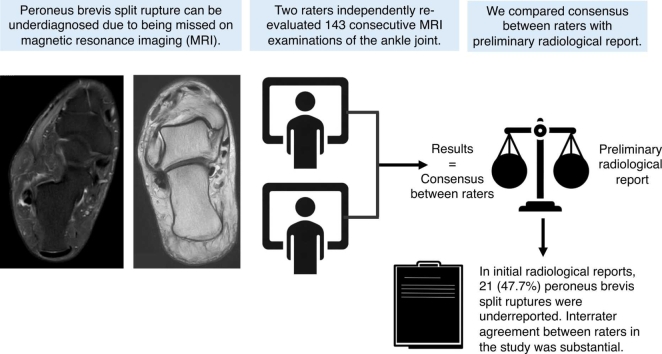

## 1 Introduction

The injury to the peroneus brevis tendon can limit physical activity. Peroneus brevis tendon injuries are often missed following an ankle sprain. Symptoms include stress-induced pain extending from below the malleolus to the proximal area along the peroneal tendons, ankle instability, and soft-tissue swelling. If conservative treatment fails, surgical intervention can be recommended [1].The precise frequency of split rupture of the peroneus brevis is not known. Some articles suggest that the incidence is probably 30–60 %[2], however the true incidence is not known, since clinical symptoms are nonspecific and may overlap with lateral ligament ruptures. The split rupture of the peroneus brevis is more common than split of the peroneus longus [3], [4]. It is known that most peroneus brevis split ruptures occur in the groove on the lateral malleolus, and transverse injuries typical of other tendons are rare in the peroneus brevis tendon [5], [6]. Peroneus brevis split rupture may coexist with or result from instability if the anterior talofibular and calcaneofibular ligament are torn. These ligaments anatomically connect to the peroneal retinaculum stabilizing the peroneal tendons in the groove on the lateral malleolus [7], [8].

A peroneus brevis split rupture can lead to ankle dysfunction and recurrent ankle varus sprains and damage the articular surface of the ankle joint, thus increasing the risk of osteoarthritis [9]. Patients typically experience pain and swelling in the ankle. After enduring discomfort for many weeks, patients often seek healthcare. Imaging, which is expensive, may limit access to diagnosis. Even with MRI, peroneus brevis split ruptures can be missed, delaying diagnosis and increasing complications. Underreporting these ruptures can result in more complications, pain, and higher healthcare costs.

After a period of nonspecific pain and discomfort on the lateral side of the ankle joint, the patient is referred for ultrasound or magnetic resonance imaging (MRI). The International Olympic Committee (IOC) [10] emphasizes the importance of preventing sports injuries, including damage to the peroneal tendons. The role of MRI is crucial in early detection of pathology, enabling prompt treatment. However, clinical, and radiological diagnosis of peroneal tendon injuries is challenging, and may lead to missed diagnoses. Unfortunately, there are no studies on how many peroneus brevis injuries are missed on MRI examinations.

## 2 Aim

The aim of the study was to investigate how often peroneus brevis split rupture is underreported in patients with lateral ankle pain.

## 3 Material and methods

### 3.1 Study design

The retrospective cohort single center study.

### 3.2 Flow

This study re-evaluated 143 consecutive magnetic resonance imaging (MRI) ankle examinations conducted in our region during 2021 due to chronic ankle pain. Chronic ankle pain was defined as pain persisting for more than 8 months[11], [12], [13]. Two experienced musculoskeletal radiologists, each with over five years of expertise, independently reviewed the examinations, focusing on the peroneus brevis tendon. The patients were included in the study until a power of 0.8 was achieved, alfa 0.05.

### 3.3 Inclusion criteria

Chronic lateral ankle pain lasting more than 8 months. The posterolateral part was defined as pain posterior to the lateral malleolus. The eight-month duration was chosen based on clinical practice and previous literature, which suggest that postero-lateral ankle pain persisting for this length of time warrants further radiological investigation [11], [12], [13].

### 3.4 Exclusion criteria

Trauma within the last 3 months, total rupture of the peroneus brevis tendon, postoperative changes, artifacts, previous fibula fractures in the examination area.

### 3.5 Examinations

The MRIs were performed using a 1.5 and 3 T scanner with an ankle coil, employing at least oblique proton density-weighted sequences with fat suppression and axial proton density sequences. The most common protocol included, in addition to the mentioned sequences, sagittal section T1-weighted turbo spin echo, coronal section T2-weighted turbo spin echo, and sagittal section proton density-weighted turbo spin echo. The patient was in a supine position, a dedicated ankle coil was used.

### 3.6 Raters

Two musculoskeletal radiologists, one with 12 years of experience and the other with 8 years of experience, evaluated MRI examinations on the dedicated radiological station. They assessed the presence or absence of incomplete or complete peroneus brevis split rupture. The final result was consensus between the raters and will be used as reference.

### 3.7 Initial radiological reports

The raters assessed the original clinical radiological reports to determine if they indicated the presence or absence of incomplete or complete peroneus brevis split rupture.

### 3.8 Definitions of variables

**Fig. 1:**
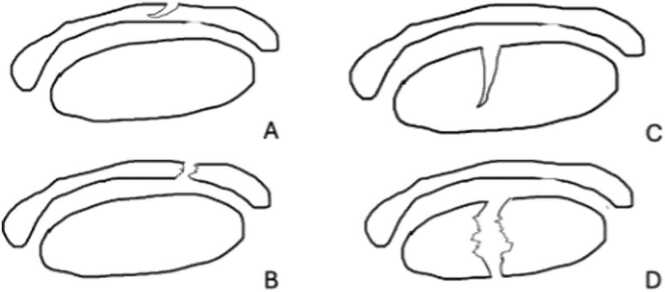
The peroneus split ruptures in transverse cross sections. A – incomplete peroneus brevis split rupture. B – complete peroneus brevis split rupture. C – incomplete peroneus longus split rupture. D – complete peroneus longus split rupture (Figure prepared by PS).

Raters evaluated the presence or absence of incomplete or complete peroneus brevis split rupture. Split ruptures (Fig. 1) were classified based on their appearance as complete (tendon split into two or more pieces) or incomplete (visible defect on the tendon surface on at least two successive images).

### 3.9 Statistical analysis

Agreement between the initial radiological report and evaluation by two raters will be calculated using Gwet’s AC1 coefficients[14] on AgreeStat® 360 and interpreted using the Landis and Koch[14], [15].

### 3.10 Ethic review

The Swedish Ethical Review Authority approved the study (number 2020–06–177 and 2021–05447).

## 4 Result

In the analyzed MRI examinations, we identified 48 patients, n=21 (43.8 %) females and n=27 (56.3 %) males (p>.05). The mean age was 52 ± 11 years, the average weight was 78.3 kg, height 1.8 m and BMI 24.0, Table 1. The right ankle was examined in 27 cases, the left one in 21 cases (p>.05).

**Table 1:** Population in the study.

	Weight (kg)	Height (m)	BMI
Average	78.3	1.8	24.0
Maximum	110.0	2.0	35.9
Minimum	50.0	1.6	19.4
Standard deviation	15.3	0.1	3.4

### 4.1 Evaluation by raters

**Fig. 2:**
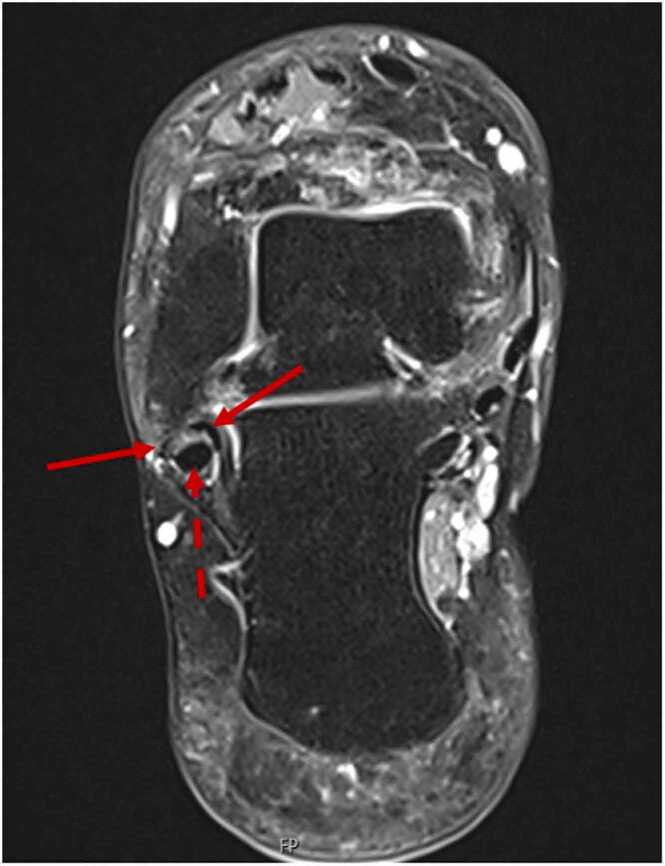
A 44-year-old patient with pain behind the lateral malleolus pain persisting for more than 8 months. The MRI scan, transverse proton density with fat suppression, shows a complete split rupture of the peroneus brevis (solid red arrow). The peroneus longus tendon is normal and is marked with a dashed arrow.

**Fig. 3:**
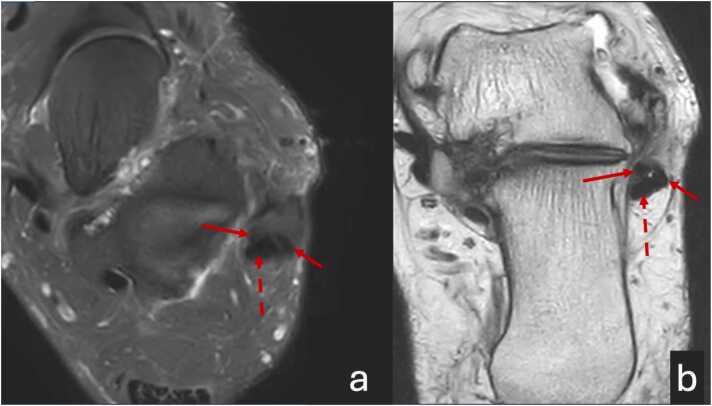
A 52-year-old patient with pain behind the lateral malleolus for approximately 12 months. The MRI scan: a - transverse proton density with fat suppression, b - T2-weighted image. The MRI shows a complete split rupture of the peroneus brevis (solid red arrow). The peroneus longus tendon is normal and is marked with a dashed arrow.

Raters identified 30 patients with complete peroneus brevis split rupture, 14 with incomplete peroneus brevis split rupture (Table 2, Fig. 2, Fig. 3). The Gwet’s AC1 coefficients showed that the agreement between raters in the study was 0.716 (standard error 0.082), 95 % CI (0.551, 0.881), p<.001 which is substantial according to Landis and Koch[14], [15].

**Table 2:** Frequency of peroneus brevis split rupture according to consensus between raters and the initial radiological reports.

		Consensus between raters		Initial radiological reports	
		n	%	n	% in relation to consensus
Peroneus brevis	Complete	30	68.2 %	Data not available due to insufficient and inconsistent reporting.	
	Incomplete	14	31.8 %		
	*Total*	44		23	52.3 %

### 4.2 Initial radiological reports

Due to inconsistent nomenclature used in the analyzed radiological reports, it was difficult to determine whether the radiologist reported complete or partial split rupture (Table 2). For study purpose, we included the sum of tendon split tears reported in the initial radiological report. Initially in the radiological reports reported n=23 (52.3 %) cases of peroneus brevis split rupture and two peroneus longus split rupture were reported, Table 2.

Because of different terminology used in the original radiological report we needed to simplify the scale to tendon split vs. no tendon split. The agreement between two raters and the initial radiological report was evaluated using Gwet’s AC1 coefficients showed that the agreement between raters and initial reports was 0.401 (standard error 0.070), 95 % CI (0.261, 0.541), p<.001 which is moderate according to Landis and Koch[14], [15].

## 5 Discussion

The most significant finding in our study was that approximately half of the split ruptures of the peroneus brevis tendon may be missed on the initial radiological report. The agreement between musculoskeletal radiologists evaluating MRI in this study was higher compared to the agreement between the radiologist reporting the clinical MRI examinations and the consensus between musculoskeletal radiologists in the study. We decided to use Gwet’s AC1 because AC1 statistic can overcome the limitations of kappa, which is sensitive to trait prevalence and rater classification probabilities called "kappa paradox"[16]. One of the main advantages is that Gwet’s AC1 is less affected by the prevalence and marginal distributions of the data. Cohen's Kappa can give misleadingly low agreement values when the data is imbalanced or when the categories are rare, which is known as the "kappa paradox."[17] This can occur even when the percentage of agreement between raters is high. In contrast, Gwet’s AC1 provides a more stable and reliable measure of inter-rater agreement under these conditions, making it a more robust choice for many studies. By using Gwet’s AC1, researchers can obtain a more accurate reflection of the true agreement between raters, leading to better-informed conclusions and more reliable results. AC1 provides a more robust measure of interrater reliability [18].

This may be because peroneus split rupture is difficult to diagnose both clinically [19], which may affect the completeness of clinical data in referrals [20]. Patients with peroneus split rupture often also have injuries to the anterior talofibular ligament and calcaneofibular ligament [12], [21]. These ligaments, through their connections [7], [8] with the superior trochlear retinaculum, can significantly impact the injuries to the latter. The function of the peroneal retinaculum is to stabilize the peroneal tendons. If this structure does not function properly, instability of the peroneal tendons may occur, leading to structural changes and, consequently, peroneus split rupture [21]. In our study, we identified several peroneus longus split ruptures; however, their number was significantly smaller than peroneus brevis split ruptures, making drawing conclusions or conducting statistical analyses on such a small group unfeasible. We believe that a separate study to assess peroneus longus split rupture would be worthwhile.

The process of peroneus brevis tendon degeneration is not entirely understood [22] but it can be long-term and multi-stage [23] and can be chronic or acute [19], [24]. It begins with changes within the tendon, which can gradually lead to longitudinal damage[4] along one surface of the tendon. The peroneus brevis split is more often than peroneus longus split rupture which is in according to our study [3], [4]. This results in incomplete peroneus brevis split rupture, defined in our study as incomplete. If such damage remains undetected and degeneration progresses, it may lead to damage extending across the entire thickness of the tendon, defined in our study as complete split. In the initial radiological reports, different terms were used to describe observed complete and incomplete ruptures, which made it impossible to assess whether the radiologist reported incomplete or complete splits. We were forced to simplify the scale (presence of split – absence of split) to evaluate the agreement between the raters and the initial radiological report. This indicates the need for further education of radiologists regarding reporting ankle injuries, as peroneus brevis split can be complete or incomplete.

Another potential reason why such a large number of peroneus brevis split ruptures may be missed is the static nature of magnetic resonance imaging (MRI) and anatomical conditions. The specificity of MRI in diagnosing peroneus brevis split rupture is about 44 %, while the sensitivity is 99 % [3]. The patient lies in the supine position, and the ankle joint is positioned in a dedicated coil, resembling a boot. Most peroneus brevis split ruptures occur at the level of the lateral malleolus. Longitudinally spited peroneus brevis is located between the fibula, whose cortical layer has low signal intensity on MRI, and the peroneus longus tendon, which is wide, strong, and has low signal intensity. If the split fragments of the peroneus brevis tendon are not sufficiently separated to be distinguished from each other, they may be missed. Thus, two small split up fragments of the peroneus brevis are wedged between the fibula and the peroneus longus tendon, making their perception difficult. Additionally, the synovial sheath at this level is narrower due to structures passing at this level compared to the width below the lateral malleolus.

In part, a significant portion of missed peroneus brevis split ruptures may be due to the experience of radiologists and low agreement in ankle MRI reporting[21]. We do not have access to data regarding the experience of the radiologists who initially assessed the MRI scans. However, we demonstrated that the agreement between musculoskeletal radiologists' assessments was greater. This suggests that the high degree of subspecialization in musculoskeletal radiology is an important factor that could potentially improve diagnosis and, consequently, clinical outcomes.

In addition to the varying experience levels of radiologists, the results of our study may also depend on the methodology employed. Raters in our study were tasked with assessing peroneal tendons, which to some extent introduces bias compared to clinical ankle assessments, where the radiologist must evaluate all structures in the examination area. Furthermore, radiologists evaluating the scans likely work under greater time pressure than those assessing peroneal tendons in the current study.

Diagnosing a peroneus brevis split can be clinically challenging. While the value of MRI in diagnosis is well recognized [3], no prior studies have examined how many split ruptures might be missed in clinical settings. Our findings indicate that detection of peroneus brevis splits can be significantly improved when more experienced raters evaluate the examinations. Our musculoskeletal radiologists identified about 50 % more split ruptures. Based on our study, we recommend that orthopedic specialists consider having "negative" ankle MRIs re-evaluated by more experienced raters if there is a high clinical suspicion of a split rupture.

Our study results indicate that when musculoskeletal radiologists evaluate scans, more peroneus brevis splits are detected. In the future, when designing similar studies, it is important to include specialized raters among the evaluators. Further studies should also consider using intraoperative findings as references, allowing for the application of different definitions of split rupture. Additionally, future research should take into account new imaging techniques, including tendon-specific sequences and dynamic imaging [25]. The application of two imaging methods, such as ultrasound and MRI, could also be utilized in future studies.

Limitations of the study. We see several limitations of this study. The first is its retrospective nature, which is related to the difficulty of clinically detecting peroneus brevis split rupture. Our study is the single center study. The lack of surgical correlation may somewhat underestimate the true frequency of peroneal split rupture occurrence. Thus, our study suggests that missed peroneal split ruptures is at least approximately 50 %. Given the chance for surgical comparison, the frequency might be slightly higher since direct surgical examination is more sensitive. In clinical practice, unfortunately, this is not possible because not all peroneus brevis split ruptures are treated surgically and not all patients would like to be operated. Inconsistent nomenclature in the initial radiological reports hindered assessment of the agreement between the reported complete and incomplete splits between radiologist and raters in this study. If we could have asked radiologist what type of split they reported, it would have been possible to compare the actual agreement between researchers and radiologists in reporting peroneus brevis incomplete and complete splits. Excluding patient groups with recent trauma, fractures may impact the study's external validity.

## 6 Conclusion

Peroneus brevis split rupture is underreported on MRI scans of patients with lateral ankle pain. While there is high agreement among radiologists routinely reporting ankle MRI, the consistency in assessing peroneus brevis split rupture between the initial radiological report and musculoskeletal radiologists is low. These findings indicate the importance of educating radiologists interpreting ankle MRI scans and improving examination methodology. In cases of strong clinical suspicion of a peroneus brevis split rupture with a negative MRI result, the clinician should request a re-review of the scan by a musculoskeletal radiologist.

## Ethics approval and consent to participate

The Swedish Ethical Review Authority approved the study (number 2020–06–177 and 2021–05447).

## Authors' contributions

PS conceived the idea of this study. KBD, PS analyzed MRI examinations. KBD and PS wrote the first draft of the manuscript. KBD and PS selected appropriate MRI figures and prepared figures. KBD, PS, DM, AA and KNH analyzed the result. All authors read and approved the final manuscript.

## Funding

This project received no funding.

## CRediT authorship contribution statement

**Katarzyna Bokwa-Dąbrowska:** Writing – review & editing, Writing – original draft, Visualization, Validation, Supervision, Software, Resources, Methodology, Investigation, Formal analysis, Data curation, Conceptualization. **Dan Mocanu:** Writing – review & editing, Visualization, Data curation. **Alex Alexiev:** Writing – review & editing, Visualization, Data curation. **Katarina Nilsson Helander:** Writing – review & editing, Resources, Methodology, Formal analysis. **Pawel Szaro:** Writing – review & editing, Writing – original draft, Methodology, Investigation, Formal analysis, Data curation, Conceptualization.

## Declaration of Generative AI and AI-assisted technologies in the writing process

Nothing to disclose

## Declaration of Competing Interest

The authors declare that they have no known competing financial interests or personal relationships that could have appeared to influence the work reported in this paper.

## Data Availability

Yes.
